# Discovery of Two Novel Negeviruses in a Dungfly Collected from the Arctic

**DOI:** 10.3390/v12070692

**Published:** 2020-06-27

**Authors:** Gang Lu, Zhuang-Xin Ye, Yu-Juan He, Yan Zhang, Xin Wang, Hai-Jian Huang, Ji-Chong Zhuo, Zong-Tao Sun, Fei Yan, Jian-Ping Chen, Chuan-Xi Zhang, Jun-Min Li

**Affiliations:** State Key Laboratory for Managing Biotic and Chemical Threats to the Quality and Safety of Agro-Products, Key Laboratory of Biotechnology in Plant Protection of Ministry of Agriculture and Zhejiang Province, Institute of Plant Virology, Ningbo University, Ningbo 315211, China; lugang@nbu.edu.cn (G.L.); yzx244522794@163.com (Z.-X.Y.); 15940518901m@sina.cn (Y.-J.H.); zhangxiaoli1028@163.com (Y.Z.); wangzhichaowork@163.com (X.W.); huanghaijian@nbu.edu.cn (H.-J.H.); zhuojichong@nbu.edu.cn (J.-C.Z.); ztaosun@gmail.com (Z.-T.S.); yanfei@yeah.net (F.Y.)

**Keywords:** negevirus, insect specific virus, virus evolution, small interference RNA

## Abstract

Negeviruses are a proposed group of insect-specific viruses that can be separated into two distinct phylogenetic clades, Nelorpivirus and Sandewavirus. Negeviruses are well-known for their wide geographic distribution and broad host range among hematophagous insects. In this study, the full genomes of two novel negeviruses from each of these clades were identified by RNA extraction and sequencing from a single dungfly (*Scathophaga furcata*) collected from the Arctic Yellow River Station, where these genomes are the first negeviruses from cold zone regions to be discovered. Nelorpivirus dungfly1 (NVD1) and Sandewavirus dungfly1 (SVD1) have the typical negevirus genome organization and there was a very high coverage of viral transcripts. Small interfering RNAs derived from both viruses were readily detected in *S. furcata*, clearly showing that negeviruses are targeted by the host antiviral RNA interference (RNAi) pathway. These results and subsequent *in silico* analysis (studies) of public database and published virome data showed that the hosts of nege-like viruses include insects belonging to many orders as well as various non-insects in addition to the hematophagous insects previously reported. Phylogenetic analysis reveals at least three further groups of negeviruses, as well as several poorly resolved solitary branches, filling in the gaps within the two sub-groups of negeviruses and plant-associated viruses in the *Kitaviridae*. The results of this study will contribute to a better understanding of the geographic distribution, host range, evolution and host antiviral immune responses of negeviruses.

## 1. Introduction

Insect-specific viruses (ISVs) are those viruses that are confined exclusively to insects and which are unable to replicate in vertebrates or vertebrate cells [1]. ISVs have been largely overlooked for a long time because they do not cause disease in vertebrate hosts and usually have no economic impact on animals or plants. The recent rise of next generation sequencing and metagenomics has led to the discovery of a growing number of novel ISVs [2]. These have mostly been discovered in hematophagous insects, especially mosquitoes, as a result of a research into the risks that mosquito-borne viruses pose to the health of humans and domesticated animals [3]. Interestingly, the majority of ISVs are phylogenetically related to the classical arthropod-borne viruses (arboviruses) transmitted by mosquitoes. It has therefore been hypothesized that ISVs might be the ancestors of arboviruses and can act as natural regulators of the infection, replication and transmission of arboviruses [3,4]. Most ISVs can be recognized as members of the families of *Bunyaviridae*, *Flaviviridae*, *Mesoniviridae*, *Reoviridae*, *Rhabdoviridae*, *Togaviridae*, or a novel group described as negeviruses [1,5].

Negeviruses are ISVs with a non-segmented, positive-sense single strand RNA genome of about 9 to 10 kb and have been separated. They are usually assigned into two distinct phylogenetic clades named in the literature as Nelorpivirus and Sandewavirus [6] although these are not yet formally recognized taxa. Negevirus genomes are polyadenylated at their 3′ terminus and encode three open reading frames (ORFs) separated by intergenic regions. The largest, ORF1, is predicted to encode a polyprotein containing four domains involved in RNA replication: viral methyltransferase (vMet) domain; RNA ribosomal methyltransferase (FtsJ) domain; viral helicase (Hel) domain; and RNA-dependent RNA polymerase (RdRp) domain. ORF2 and ORF3 are believed respectively to encode a structural protein containing a predicted glycoprotein domain and a membrane protein, although little experimental evidence has been provided [5,7,8]. A previous study has suggested that the Ochlerotatus caspius negevirus (OCNV) can replicate in C6/36 cells (established from macerates of *Aedes albopictus* larvae) through the use of a double strand RNA (dsRNA) intermediate, which can be formed as early as 3 h after infection [9]. More recent small RNA analysis indicated that these dsRNA intermediates of nege-like viruses might trigger the small interfering RNA (siRNA) mediated antiviral pathway in the host insect [10].

The first study, in which six negeviruses were discovered from pools of mosquitoes and phlebotomine sand flies from 1977 to 2008, were collected in North and South America, Africa and Asia [5]. Since then, more negeviruses have been discovered worldwide, including the Tanay virus (TANAV) from the Philippines [11], the Okushiri virus from Japan [7], new strains of TANAV from China [12], the Goutanap virus from Côte d’Ivoire [6] in Africa, the Castlerea virus from Australia [13], the OCNV and Culex univittatus negevirus from Portugal [9], and the Wallerfield virus (WALV) from Brazil, Trinidad and Colombia [14,15]. These negeviruses have been from insects in tropical, sub-tropical and temperate regions between the latitudes 42° N and 42° S [16]. Most negeviruses have been identified from mosquito hosts, including nine genera within the family *Culicidae*, order *Diptera* [15]. Other potential insect hosts of negeviruses have included sandflies, bees, aphids, and fruitflies and they have also been found in some other animals such as nematodes, shrimps and mites [5,17,18,19], indicating that negeviruses may have a much broader host range than originally thought. However, the diversity of negeviruses in hosts other than hematophagous insects have not been well evaluated.

Phylogenetic analysis indicates that negeviruses are distantly related to plant-infecting viruses classified in the genera *Cilevirus*, *Higrevirus* and *Blunevirus* in the family *Kitaviridae* [15]. It has therefore been suggested that negeviruses and viruses in the family *Kitaviridae* might have originated from a common arthropod-infecting ancestral virus [15,20] although the potential evolutionary pathway leading to host expansion has not been clearly elucidated.

In this study, the complete genomes of two novel negeviruses have been identified and characterized from a single non-hematophagous dipteran insect (dungfly, *Scathophaga furcata*) collected from the arctic. This expands the known host range and geographical distribution of negeviruses. Subsequent phylogenic analysis using other novel nege-like viruses from various insect hosts can provide new insights into the diversity of these viruses and the relationship between nege-like viruses and plant viruses. In addition, virus-derived small interfering RNAs (vsiRNA) were comprehensively investigated, suggesting that the host siRNA-mediated antiviral pathway might be involved actively against negeviruses.

## 2. Materials and Methods

### 2.1. Sample Preparation and RNA Extraction

A single dungfly-like adult insect was collected on July 2019 from Arctic Yellow River Station (Latitude: 78.9167, Longitude: 11.9333). The insect was captured alive and immediately transferred to RNA keeper tissue stabilizer (Vazyme, Nanjing, China) at low temperature (4 °C) and sent to our laboratory for RNA extraction. Total RNAs were extracted using TRIzol reagent (Invitrogen, Waltham, MA, USA) following the manufacturer’s instructions and subdivided to provide samples for transcriptome (approximately 2 μg), small RNA (sRNA) (approximately 5 μg) and virus genome Sanger sequencing (approximately 5 μg).

### 2.2. Transcriptome and sRNA Sequencing

For transcriptome sequencing, ribosomal RNA (rRNA) was first removed from the total RNA using Ribo-Zero Gold rRNA Removal Kit (Illumina, San Diego, CA, USA) before preparing the sequencing library. Paired-end (150 bp) sequencing of the RNA library was performed on the Illumina HiSeq 4000 platform (Illumina, San Diego, CA, USA) by Novogene (Tianjin, China). The transcriptome reads were quality trimmed and assembled de novo using the Trinity software (Version 2.8.5) with default parameters [21].

The cDNAs of the sRNA library were prepared using the Illumina TruSeq Small RNA Sample Preparation Kit (Illumina, San Diego, CA, USA). sRNA sequencing was performed on an Illumina HiSeq 2500 by Novogene (Tianjin, China). Preliminary treatment of sRNA raw data (removal of adapter, low quality, and junk sequence) was carried out as described previously [22].

### 2.3. Host Insect Identification

To accurately identify the dungfly species, the assembled contigs from the transcriptome were compared using Blastn with all the available cytochrome oxidase subunit 1 (COI) barcode records from the Barcode of Life Data (BOLD) Systems (http://www.boldsystems.org/) and the National Center for Biotechnology Information (NCBI) nucleotide (nt) database. The identified COI sequence of the dungfly was further confirmed by Sanger sequencing and submitted to GenBank with the accession number MT072894.

### 2.4. Virus Discovery and Confirmation by Reverse Transcription-PCR (RT-PCR)

To identify nege-like viral contigs, the assembled transcriptome contigs were compared to a nucleotide/protein database comprising representative negeviruses (Supplementary Table S1) downloaded from GenBank using BLAST+ (Version 2.9.0) and DIAMOND (Version 0.9.28.129). The e-value threshold for the comparisons was set at 2 × 10^−10^. The candidate nege-like virus contigs were then extracted using home-made perl script based on the significance of the e-value and the matched length of the contig. To eliminate false positives, the candidate nege-like virus contigs were further compared with the entire NCBI nucleotide (NT) and non-redundant (NR) protein databases. RT-PCR was then performed followed by Sanger sequencing to confirm the presence of the two full nege-like virus contigs using the method described previously [22]. The primers used for RT-PCR are listed in Supplementary Table S2.

### 2.5. Determination of Viral Genome Termini and Transcript Abundance

To obtain the full length of the two identified negeviruses in the insect sample, the extreme 5′ and 3′ terminal sequences were determined by rapid amplification of cDNA ends (RACE) using the SMARTer^®^ RACE 5′/3′ kit (Takara, Beijing, China). After total RNA isolation, first-strand cDNA synthesis was performed to obtain 5′-RACE-ready and 3′-RACE-ready cDNA according to the manufacturer’s instructions. Touchdown PCR was performed to amplify RACE products using 5′ or 3′ GSPs (gene-specific primers) and UPM (Universal Primer A Mix). The PCR products were then cloned into the pMD19-T vector (Takara, Beijing, China) and further verified by Sanger sequencing. The primers used for RACE are listed in Supplementary Table S2. Based on the Blast search results, the two identified negeviruses were named Nelorpivirus dungfly1 (NVD1) and Sandewavirus dungfly1 (SVD1) and the sequences were submitted to GenBank with the respective accession numbers MT344120 and MT344121.

To investigate the transcript abundance and coverage of the two identified negeviruses, the adaptor- and quality-trimmed reads of the transcriptome were mapped back to the whole genome of NVD1 and SVD1 using Bowtie2 [23] and Samtools [24]. The coverage of the aligned reads to the virus genomes was further visualized using the Integrated Genomics Viewer [25].

### 2.6. Genome Annotation

Open reading frames (ORFs) of NVD1 and SVD1 were predicted by the ORF Finder online server (https://www.ncbi.nlm.nih.gov/orffinder/). Predicted conserved protein domains were identified using the Conserved Domain Database server [26]. Potential glycosylation sites were predicted using the NetNGlyc 1.0 Server (http://www.cbs.dtu.dk/services/NetNGlyc/). Transmembrane domains were analyzed by the TMHMM server v. 2.0 (http://www.cbs.dtu.dk/services/TMHMM/).

### 2.7. Small RNA Analysis

To identify siRNAs derived from NVD1 and SVD1, clean sRNA reads 18- to 30-nt long were extracted and collapsed using FASTX-Toolkit (http://hannonlab.cshl.edu/fastx_toolkit/). The processed reads were mapped to the assembled full genomes of NVD1 and SVD1 using Bowtie software allowing for zero mismatches [27]. Downstream analysis for the mapped vsiRNA was performed with custom perl scripts and Linux bash scripts, including size distribution of vsiRNA, vsiRNA distribution along the corresponding viral genome, and 5′ terminal nucleotide preference of 21 nt long vsiRNAs.

### 2.8. Prevalence of Nege-Like Viruses Were Investigated in Invertebrates

The prevalence of possible nege-like viruses in other hosts was investigated using the public Expression Sequence Tag (EST) and Transcriptome Shotgun Assembly (TSA) databases of NCBI. The putative protein sequences of representative known negeviruses (Supplementary Table S1) together with NVD1 and SVD1 were used as query, searching against the EST and TSA databases using tblastn. The potential novel nege-like viral contigs were then compared with the entire NCBI NT and NR databases to eliminate false positives.

### 2.9. Phylogenetic Analyses

Phylogenetic analysis used the amino acid sequences of the predicted RNA dependent RNA polymerase (RdRp) region of the newly identified nege-like viruses from this study, together with some previously described negeviruses from various hosts and plant viruses of the related families *Kitaviridae* and *Virgaviridae.* Sequences were obtained from NCBI, aligned using Muscle (Version 3.8.31) [28] and analyzed using the Maximum likelihood (ML) algorithm and the Jones-Taylor-Thornton (JTT) substitution model to construct a phylogenetic tree in MEGA X with 1000 bootstrap replications [29].

## 3. Results

### 3.1. Negeviruses Identified in Dungfly

A total of 84,181 contigs were generated from de novo assembly of the clean RNA-seq reads (38,056,018). A Blast search among the COI sequences confirmed that the dungfly was *Scathophaga furcata* (*Diptera*: *Scathophagidae*). A BlastX search against the proteins of representative negeviruses suggested the presence of two potential new negeviruses. The nearly complete genomes of both were identified in the insect. One contig of 9212 nt was identified as a nelorpivirus (NVD1), and was most similar to the Loreto virus (LRV, YP_009351835.1) with protein sequence identities of 69%. The second contig (8858 nt), representing SVD1, was most similar to the sandewavirus Andrena haemorrhoa nege-like virus (AHNLV, YP_009553581.1) with identities of 55%. A Blastn search against the NCBI NT database did not find any other sequences closely related to either NVD1 or SVD1, indicating that the two viruses are probably new negeviruses. The full genome sequences of both viruses were then verified by RT-PCR followed by Sanger sequencing and RACE to determine their 5′ and 3′ termini.

### 3.2. Genome Organization of NVD1 and SVD1

**Figure 1:**
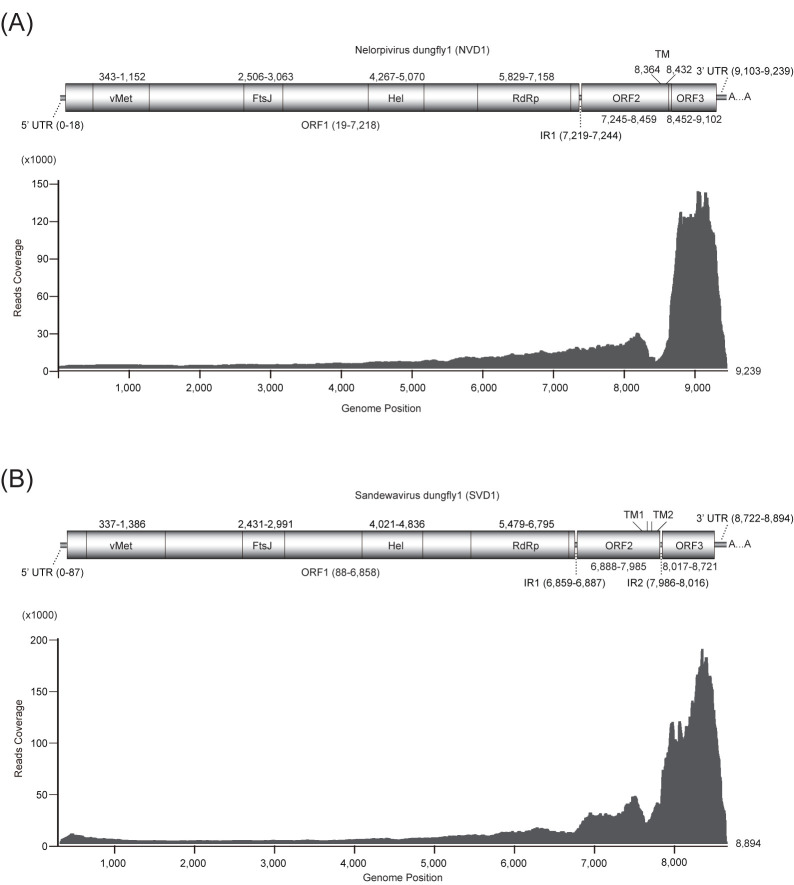
Genome organization and transcriptome raw read coverage of Nelorpivirus dungfly1 (**A**) and Sandewavirus dungfly1 (**B**). vMet, viral methyltransferase domain; FtsJ, RNA ribosomal methyltransferase domain; Hel, viral helicase domain; RdRp, RNA-dependent RNA polymerase domain; UTR, untranslated region; IR, intergenic region; TM, transmembrane domain; TM1 region, 7743–7811; TM2 region, 7872–7940.

The full-length sequences of NVD1 and SVD1 were respectively 9239 and 8894 nt long excluding the polyA tail. The predicted genome organization of both viruses is typical of that reported for negeviruses with three major ORFs (Figure 1). ORF1 has the four conserved domains of the replication polyprotein (vMet, FtsJ, Hel and RdRP). N-glycosylation sites were predicted in ORF2 at amino acid positions 105, 138, 170, and 175 (NVD1), and positions 124, 161, 248, and 278 for SVD1. One (NVD1) or two (SVD1) transmembrane domains are present at the C-terminus of ORF2. While previously reported negeviruses and SVD1 have short intergenic regions between each of the ORFs, the ORFs 2 and 3 of NVD1 unusually overlap by 8 nt and are in different frames (Figure 1A). Re-alignment of the RNA-seq reads to the reconstructed complete genomes of NVD1 and SVD1 show a very high mean coverage (6858× for NVD1 and 8857× for SVD1), suggesting that the viruses replicate very efficiently in their host. Viral transcripts were very highly elevated in the 3′ region of the genome of both viruses (Figure 1).

### 3.3. NVD1 and SVD1 Are Targeted by the Host siRNA-Based Antiviral RNAi Pathway

**Figure 2:**
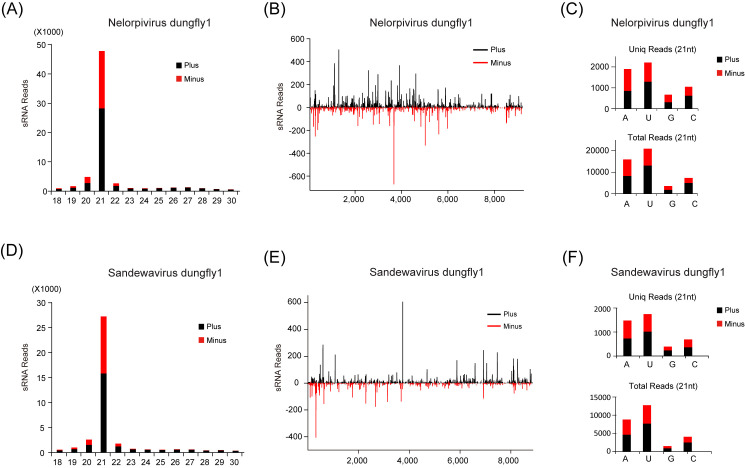
Profiles of virus-derived small interfering RNAs (vsiRNAs) of Nelorpivirus dungfly1 (NVD1) and Sandewavirus dungfly1 (SVD1). (**A**) Size distribution of NVD1 sRNAs. (**B**) Distribution of NVD1-derived sRNA along the corresponding viral genome. (**C**) 5′ terminal nucleotide preference of sRNAs 21 nt long derived from NVD1. (**D**) Size distribution of SVD1 sRNAs. (**E**) Distribution of SVD1 derived sRNA along the corresponding viral genome. (**F**) 5′ terminal nucleotide preference of sRNAs 21 nt long derived from SVD1. Color coding indicates viral sRNAs derived, respectively, from the sense (plus) and antisense (minus) genomic strands.

siRNA-based RNA silencing is an important antiviral pathway in insects and is usually associated with the accumulation of vsiRNAs as viral RNA is degraded in a sequence-specific manner [30]. To better understand siRNA-based antiviral pathways in dungfly in response to negeviruses, we conducted a computational analysis of vsiRNAs in the sRNA library of *S. furcata*. A large number of siRNAs (18 nt–30 nt) derived from the two negeviruses were identified. A total of 68,717 sRNA reads (16,995 unique) mapped perfectly to the assembled genome of NVD1, accounting for 0.25% (1.65% unique) of the whole sRNA library. The corresponding vsiRNA reads for SVD1 totaled 38,672 (11,053 unique), accounting for 0.14% (1.07% unique) of the library. Most of these vsiRNAs were 21 nt long (69.5% and 70.1% of the totals for NVD1 and SDV1, respectively) and they were equally derived from the sense and antisense strands of the viral genomic RNA (Figure 2A,D), which are similar to the recent report [10]. The vsiRNAs were derived from the entire genome of both viruses including the untranslated regions, but there were notable asymmetric hotspots on both strands, suggesting that these regions might be preferential targets of the host immune system (Figure 2B,E). The viral siRNAs of both viruses had a strong A/U preference in their 5′ terminal nucleotide (Figure 2C,F), which is typical of vsiRNAs from various organisms, including insects [22,31]. These characteristics provide strong evidence that the antiviral RNAi pathway of dungfly is actively involved in response to negevirus infection.

### 3.4. The Presence of Further Nege-Like Virus Sequences in Public Databases Suggests That They Occur in Many Different Insects

Negeviruses are well-known for their wide geographic distribution and broad host range but most of the well-described ones have been isolated from hematophagous insects such as mosquitoes and sandflies [3,15]. The identification of NVD1 and SVD1 in a different type of insect and from a much colder environment prompted a search for other, potentially new, nege-like viral sequences, within the current public databases. Seven potentially new negeviruses were identified in the TSA database originating from firefly, flower thrips, sucking bugs, and various fruit fly species, suggesting a diversity and prevalence of insect hosts for nege-like viruses in nature (Table 1). In addition, reanalysis of previous virome studies confirmed that the hosts of negeviruses are broader than insects (Table 2). Within *Insecta*, and in addition to the *Diptera*, hosts of negeviruses included representatives of *Hemiptera*, *Coleoptera*, *Thysanoptera*, *Odonata*, and *Orthoptera*. Another four classes of the *Arthropoda* were also represented, including three species in *Arachnida*, one species in *Malacostraca*, one species in *Maxillopoda*, and one species in *Chilopoda*. Outside the arthropods entirely were two species in *Nematoda* and one species in *Cnidaria* [19,32]. We also have unpublished data [33] from a field investigation in 2019, which identified three nege-like viruses in whitefly (*Bemisia tabaci*) and one in grasshopper (*Metaleptea brevicornis*), and the sequences of viral RdRP regions were submitted to NCBI GenBank with accession number as listed in Table 2. It is clear that negeviruses are common in *Insecta* generally and not just in hematophagous insects.

### 3.5. Putative New Phylogenetic Clades and Host Diversity of Negeviruses in Invertebrates

**Figure 3:**
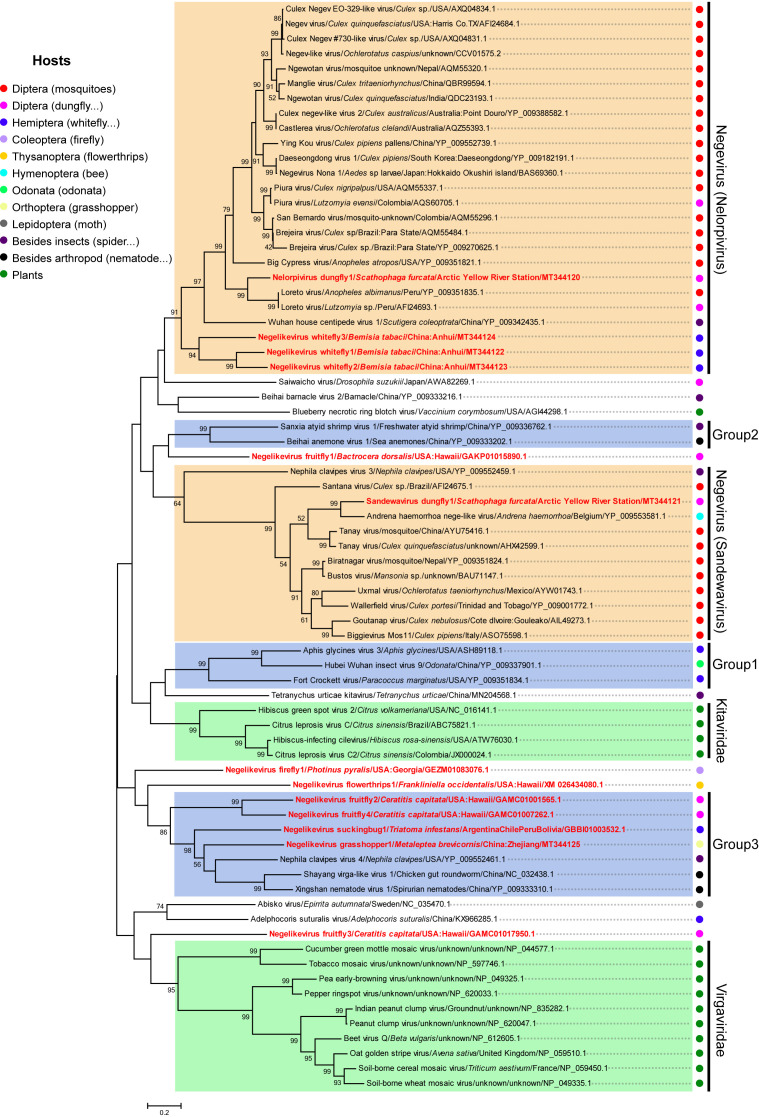
Maximum likelihood phylogenetic tree based on the amino acid sequences of the conserved RdRp domain of the newly identified nege-like viruses from this study, representative negeviruses from various hosts and plant viruses of the families *Kitaviridae* and *Virgaviridae*. Branch labels show virus name, virus host, country or origin (region) and corresponding accession numbers. Red font represents nege-like viruses identified in this study. Bootstrap values are placed over each node of the tree (when >50). Scale bars represent percentage divergence. Taxonomy of the host organism is indicated by circles of different color.

A phylogenetic tree was constructed using sequences of negeviruses from various hosts and those of closely related plant viruses. NVD1 clusters with two LRV isolates and some other insect viruses in the previously identified Nelorpivirus clade, while SVD1 falls clearly with AHNLV and other insect viruses in the Sandewavirus clade (Figure 3). The topology of the tree also confirmed the close relationship of plant viruses in the family *Kitaviridae* that was previously reported [11,15,16]. Using plant viruses of the family *Virgaviridae* as an out-group, a number of other obvious groups of nege-like viruses can also be recognized. These include a branch with three insect viruses (Abisko virus, Adelphocoris suturalis virus, and Negelikevirus fruitfly3) and at least three other groups formed with high bootstrap value (labelled Group 1, Group 2, and Group 3 in Figure 3). There are also other several poorly resolved solitary branches for nege-like viruses. Although the Nelorpivirus and Sandewavirus clades are mostly viruses from the order *Diptera* within *Insecta* (mostly mosquitoes), there are also hosts in the orders *Hemiptera* (whitefly), *Hymenoptera* (bee), and from outside insects (house centipedes and spiders) from various regions of the world. Group 2 contains two closely related nege-like viruses, Sanxia atyid shrimp virus 1 and Beihai anemone virus 1, that are from hosts classified in different phyla (*Arthropoda* and *Cnidaria*) but from a similar ecological niche.

## 4. Discussion

Since the taxon Negevirus was initially suggested, more than 100 negeviruses have been isolated worldwide, particularly from in Asia, Africa, Oceania, Europe and America [15]. All previously discovered negeviruses were from the tropical, sub-tropical and temperate regions (latitudes between 42° N and 42° S), raising the possibility that negeviruses might be affected significantly by environmental factors and not be adapted to hosts living in extreme conditions such as low temperature [16]. In this study, two new negeviruses (NVD1 and SVD1) were identified in a dungfly (*S. furcata*) collected from the arctic region (latitude 79° N), much further north than any previously described negevirus. Earlier work suggested that negeviruses from different clades might be found in the same host and geographic location [15]. This is supported and extended by our finding of two negeviruses from different clades (NVD1-Nelorpivirus, SVD1-Sandewavirus) within single individual host insect.

Negeviruses are also well-known for having a broad host range among biting Diptera, including nine genera of mosquitoes, that have been studied because of their importance to public health [3,5,15]. The viruses reported here and the subsequent in silico studies of public databases and published virome data show that the hosts of nege-like viruses are much more diverse. Known hosts now include insects belonging to the orders *Diptera*, *Hemiptera*, *Coleoptera*, *Thysanoptera*, *Hymenoptera*, *Odonata*, *Orthoptera*, and *Lepidoptera* and, interestingly, non-insect arthropods (spider, shrimp etc.) and even non-arthropod organisms (nematodes and anemone) (Table 2). These results provide strong evidence to support the previous hypothesis that the host range of negeviruses might have been greatly underestimated due to current sampling bias in favor of biting or blood-sucking arthropods [5,15]. It is clearly no longer tenable to regard negeviruses as insect-specific or mosquito-specific viruses. 

Phylogenetic studies and the discovery of Insect-specific viruses (ISV) genomic material integrated into the mosquito genome have led to the hypothesis that a number of pathogenic arboviruses may have acquired their dual host through long term adaptive evolution of former ISVs in vertebrates [20,45]. Negeviruses are genetically and evolutionarily related to plant viruses in the family *Kitaviridae* [5,6,17], and investigation of endogenous viral elements indicated that virga/nege-related viruses in insects and plants might share common viral origins [46]. Our phylogenetic analysis indicated that three nege-like viruses in the unassigned group 1 and Tetranychus urticae kitavirus are phylogenetically closer to plant viruses (*Kitaviridae*), filling the phylogenetic “gaps” between plant-associated viruses and the proposed two clades of negeviruses (Figure 3). Interestingly, a newly reported nege-like virus (Fragaria vesca-associated virus 1) isolated from a symptomatic strawberry plant also shows high homology to Aphis glycines virus 3 [47], indicating that this unassigned group might be the key connection between nege-related arthropod viruses and plant viruses. The increasing number of newly discovered nege-like viruses will surely help to clarify the uncertain relationship between nege-like viruses and associated plant viruses. The phylogenetic relationships of some nege-like viruses in the tree were incongruent with host phylogeny, especially for the viruses in the three unassigned groups (Figure 3), indicating the possibility of cross-species virus transmission in a similar ecological niche.

The high numbers of viral transcripts of NVD1 (6858×) and SDV1 (8857×) in *S. furcata* show that the viruses were infecting and propagating in the insect and were not just contaminants. Previous studies have shown that negeviruses can replicate to high viral loads in cell lines of some mosquitoes and sandflies [5,15]. In addition, dsRNA intermediates of negeviruses can be detected in mosquito C6/36 cells in early stages of infection, indicating that this replication may use a dsRNA intermediate [9]. In this study, we detected and characterized vsiRNAs derived from both NVD1 and SDV1 in *S. furcata.* These were mostly 21 nt long and were more or less equally derived from both strands of the dsRNA replication intermediates, providing clear evidence that negeviruses are targeted by the host siRNA-based antiviral RNAi pathway. It will be interesting to investigate whether negeviruses can induce similar siRNA-based antiviral immunity in non-insect hosts in the future. Several studies have shown that infection by some strains of *Wolbachia* can upregulate the mosquito’s innate immune system and then interfere with mosquito-borne virus replication by decreasing vector competence [48,49]. In addition, laboratory experiments have also indicated that insect-specific flaviviruses can downregulate the replication of heterologous flaviviruses in mosquito cells [50,51]. Since negeviruses can replicate actively in several well-known arthropod vectors of vertebrate viruses (mosquitoes, sandflies) and plant viruses (aphids, whiteflies), it will be fascinating to evaluate the impact of negevirus replication on the fitness and vector competence of these important arbovirus vectors. It is at least possible that, if true, negeviruses could have enormous potential value as biological control agents of pathogenic viruses.

## Figures and Tables

**Table 1:** Negevirus-like Contigs identified in other insects from the public database.

Tentative Name	NCBI Accession No.	Length	E-Value	Negevirus Protein	Query Start	Query End	Homology Virus	RdRP ^#^ Identity	Homology Virus Reference
Negelikevirus firefly1	GEZM01083076.1	5125	6 × 10^−154^	ORF1 (partial)	4361	1611	Hammarskog virga-like virus	40%	[34]
Negelikevirus flowerthrips1	XM_026434080.1	1791	7 × 10^−76^	ORF1 (partial)	247	1734	Buckhurst virus	38%	[35]
Negelikevirus suckingbug1	GBBI01003532.1	1488	8 × 10^−119^	ORF1 (partial)	10	1485	Hubei virga-like virus 16	46%	[19]
Negelikevirus fruitfly1	GAKP01015890.1	6984	0	ORF1 (Complete)	44	4492	Hammarskog virga-like virus	51%	[34]
Negelikevirus fruitfly2	GAMC01001565.1	10,007	0	ORF1 (Complete)	71	6526	Aedes camptorhynchus negev-like virus	62%	[36]
Negelikevirus fruitfly3	GAMC01017950.1	9925	0	ORF1 (Complete)	112	9870	Blackford virus	61%	[35]
Negelikevirus fruitfly4	GAMC01007262.1	10,264	0	ORF1 (Complete)	64	6531	Aedes camptorhynchus negev-like virus	54%	[36]

^#^ RdRP: RNA-dependent RNA polymerase.

**Table 2:** Broad host range and wild distribution of nege-like viruses in animals.

Virus Name/Tentative Name	Host Information						Location/Year	GenBank Accession No.	Reference
	Hosts/Species	Phylum	Class	Order	Family	Genus			
Nelorpivirus dungfly1	*Scathophaga furcata*	*Arthropoda*	*Insecta*	*Diptera*	*Scathophagidae*	*Scathophaga*	Arctic yellow river station/2019	MT344120	This study
Sandewavirus dungfly1								MT344121	
Negelikevirus fruitfly1	*Bactrocera dorsalis*	*Arthropoda*	*Insecta*	*Diptera*	*Tryetidae*	*Bactrocera*	Puna, Hawaii, USA/1984	GAKP01015890.1	[37]
Negelikevirus fruitfly2	*Ceratitis capitata*	*Arthropoda*	*Insecta*	*Diptera*	*Tephritidae*	*Ceratitis*	Waimanalo, Hawaii, USA/NA	GAMC01001565.1	[38]
Negelikevirus fruitfly3								GAMC01017950.1	
Negelikevirus fruitfly4								GAMC01007262.1	
Piura virus	*Lutzomyia evansi*	*Arthropoda*	*Insecta*	*Diptera*	*Psychodidae*	*Lutzomyia*	Colombia/2013	AQS60705.1	[15]
Loreto virus	*Lutzomyia sp.*	*Arthropoda*	*Insecta*	*Diptera*	*Psychodidae*	*Lutzomyia*	Peru/1977	AFI24693.1	[5]
Saiwaicho virus	*Drosophila suzukii*	*Arthropoda*	*Insecta*	*Diptera*	*Drosophilidae*	*Drosophila*	Japan/2016	AWA82269.1	[39]
Negelikevirus whitefly1	*Bemisia tabaci*	*Arthropoda*	*Insecta*	*Hemiptera*	*Aleyrodidae*	*Bemisia*	Anhui, China/2019	MT344122	[33]
Negelikevirus whitefly2								MT344123	
Negelikevirus whitefly3								MT344124	
Negelikevirus suckingbug1	*Triatoma infestans*	*Arthropoda*	*Insecta*	*Hemiptera*	*Reduviidae*	*Triatoma*	Argentina/2007, Chile/1979, Peru/2008, Bolivia/2003–2012	GBBI01003532.1	[40]
Fort Crockett virus	*Paracoccus marginatus*	*Arthropoda*	*Insecta*	*Hemiptera*	*Pseudococcidae*	*Paracoccus*	USA/2015	YP_009351834.1	[15]
Aphis glycines virus 3	*Aphis glycines*	*Arthropoda*	*Insecta*	*Hemiptera*	*Aphididae*	*Aphis*	USA/2009	ASH89118.1	[41]
Adelphocoris suturalis virus	*Adelphocoris suturalis*	*Arthropoda*	*Insecta*	*Hemiptera*	*Miridae*	*Adelphocoris*	China/2015	KX966285.1	[42]
Negelikevirus firefly1	*Photinus pyralis*	*Arthropoda*	*Insecta*	*Coleoptera*	*Lampyridae*	*Photinus*	Lawrenceville, Georgia, USA/2015	GEZM01083076.1	[43]
Negelikevirus flowerthrips1	*Frankliniella occidentalis*	*Arthropoda*	*Insecta*	*Thysanoptera*	*Thripidae*	*Frankliniella*	Oahu, Hawaii, USA/NA	XM_026434080.1	NA
Andrena haemorrhoa nege-like virus	*Andrena haemorrhoa*	*Arthropoda*	*Insecta*	*Hymenoptera*	*Andrenidae*	*Andrena*	Belgium/2015	YP_009553581.1	[18]
Hubei Wuhan insect virus 9	Odonata	*Arthropoda*	*Insecta*	*Odonata*	*-*	-	China/2013	YP_009337901.1	[19]
Negelikevirus grasshopper1	*Metaleptea brevicornis*	*Arthropoda*	*Insecta*	*Orthoptera*	*Acrididae*	*Metaleptea*	Huzhou, Zhejiang, China/2019	MT344125	[33]
Abisko virus	*Epirrita autumnata*	*Arthropoda*	*Insecta*	*Lepidoptera*	*Geometridae*	*Epirrita*	Sweden/2013	NC_035470.1	[44]
Nephila clavipes virus 3	*Nephila clavipes*	*Arthropoda*	*Arachnida*	*Araneae*	*Nephilidae*	*Nephila*	USA/2013	YP_009552459.1	[32]
Nephila clavipes virus 4	*Nephila clavipes*	*Arthropoda*	*Arachnida*	*Araneae*	*Nephilidae*	*Nephila*	USA/2013	YP_009552461.1	[32]
Tetranychus urticae kitavirus	*Tetranychus urticae*	*Arthropoda*	*Arachnida*	*Trombidiformes*	*Tetranychidae*	*Tetranychus*	China/2018	MN204568.1	NA
Sanxia atyid shrimp virus 1	Freshwater atyid shrimp	*Arthropoda*	*Malacostraca*	*Decapoda*	*Atyidae*	-	China/2014	YP_009336762.1	[19]
Beihai barnacle virus 2	Barnacle	*Arthropoda*	*Maxillopoda*	*-*	*-*	-	China/2014	YP_009333216.1	[19]
Wuhan house centipede virus 1	*Scutigera coleoptrata*	*Arthropoda*	*Chilopoda*	*Scutigeromorpha*	*Scutigeridae*	*Scutigera*	China/2013	YP_009342435.1	[19]
Shayang virga-like virus 1	Chicken gut roundworm	*Nematode*	*Secernentea*	*Ascaridida*	*Ascaridiidae*	-	China/2014	NC_032438.1	[19]
Xingshan nematode virus 1	Spirurian nematodes	*Nematode*	*Chromadorea*	*Rhabditida*	*-*	-	China/2014	YP_009333310.1	[19]
Beihai anemone virus 1	Sea anemones	*Cnidaria*	*Anthozoa*	*Actiniaria*	*-*	-	China/2014	YP_009333202.1	[19]

## Supplementary Materials

Available online: https://www.mdpi.com/1999-4915/12/7/692/s1. Table S1: Representative negeviruses used for the identification of nege-like viral contigs; Table S2: Primers used in this study.

## References

[1] Vasilakis N., Tesh R.B. (2015). Insect-specific viruses and their potential impact on arbovirus transmission. Curr. Opin. Virol..

[2] Junglen S., Drosten C. (2013). Virus discovery and recent insights into virus diversity in arthropods. Curr. Opin. Microbiol..

[3] Bolling B.G., Weaver S.C., Tesh R.B., Vasilakis N. (2015). Insect-Specific Virus Discovery: Significance for the Arbovirus Community. Viruses.

[4] Marklewitz M., Zirkel F., Kurth A., Drosten C., Junglen S. (2015). Evolutionary and phenotypic analysis of live virus isolates suggests arthropod origin of a pathogenic RNA virus family. Proc. Natl. Acad. Sci. USA.

[5] Vasilakis N., Forrester N.L., Palacios G., Nasar F., Savji N., Rossi S.L., Guzman H., Wood T.G., Popov V., Gorchakov R. (2013). Negevirus: A proposed new taxon of insect-specific viruses with wide geographic distribution. J. Virol..

[6] Kallies R., Kopp A., Zirkel F., Estrada A., Gillespie T.R., Drosten C., Junglen S. (2014). Genetic characterization of goutanap virus, a novel virus related to negeviruses, cileviruses and higreviruses. Viruses.

[7] Kawakami K., Kurnia Y.W., Fujita R., Ito T., Isawa H., Asano S., Binh N.D., Bando H. (2016). Characterization of a novel negevirus isolated from *Aedes* larvae collected in a subarctic region of Japan. Arch. Virol..

[8] Kuchibhatla D.B., Sherman W.A., Chung B.Y., Cook S., Schneider G., Eisenhaber B., Karlin D.G. (2014). Powerful sequence similarity search methods and in-depth manual analyses can identify remote homologs in many apparently “orphan” viral proteins. J. Virol..

[9] Carapeta S., do Bem B., McGuinness J., Esteves A., Abecasis A., Lopes A., de Matos A.P., Piedade J., de Almeida A.P., Parreira R. (2015). Negeviruses found in multiple species of mosquitoes from southern Portugal: Isolation, genetic diversity, and replication in insect cell culture. Virology.

[10] Zhang W., Gu Q., Niu J., Wang J.J. (2020). The RNA Virome and Its Dynamics in an Invasive Fruit Fly, Bactrocera dorsalis, Imply Interactions Between Host and Viruses. Microb. Ecol..

[11] Nabeshima T., Inoue S., Okamoto K., Posadas-Herrera G., Yu F., Uchida L., Ichinose A., Sakaguchi M., Sunahara T., Buerano C.C. (2014). Tanay virus, a new species of virus isolated from mosquitoes in the Philippines. J. Gen. Virol..

[12] Wang J., Wu J., Li N., Cao Y., He Y., Lin J., Li H. (2018). A new Tanay virus isolated from mosquitoes in Guangxi, China. Arch. Virol..

[13] O’Brien C.A., McLean B.J., Colmant A.M.G., Harrison J.J., Hall-Mendelin S., van den Hurk A.F., Johansen C.A., Watterson D., Bielefeldt-Ohmann H., Newton N.D. (2017). Discovery and Characterisation of Castlerea Virus, a New Species of Negevirus Isolated in Australia. Evol. Bioinform..

[14] Auguste A.J., Carrington C.V., Forrester N.L., Popov V.L., Guzman H., Widen S.G., Wood T.G., Weaver S.C., Tesh R.B. (2014). Characterization of a novel Negevirus and a novel Bunyavirus isolated from *Culex* (*Culex*) *declarator* mosquitoes in Trinidad. J. Gen. Virol..

[15] Nunes M.R.T., Contreras-Gutierrez M.A., Guzman H., Martins L.C., Barbirato M.F., Savit C., Balta V., Uribe S., Vivero R., Suaza J.D. (2017). Genetic characterization, molecular epidemiology, and phylogenetic relationships of insect-specific viruses in the taxon Negevirus. Virology.

[16] Zhao L., Mwaliko C., Atoni E., Wang Y., Zhang Y., Zhan J., Hu X., Xia H., Yuan Z. (2019). Characterization of a Novel Tanay Virus Isolated From *Anopheles sinensis* Mosquitoes in Yunnan, China. Front. Microbiol..

[17] Ramos-Gonzalez P.L., Dos Santos G.F., Chabi-Jesus C., Harakava R., Kitajima E.W., Freitas-Astua J. (2020). Passion Fruit Green Spot Virus Genome Harbors a New Orphan ORF and Highlights the Flexibility of the 5′-End of the RNA2 Segment Across Cileviruses. Front. Microbiol..

[18] Schoonvaere K., Smagghe G., Francis F., de Graaf D.C. (2018). Study of the Metatranscriptome of Eight Social and Solitary Wild Bee Species Reveals Novel Viruses and Bee Parasites. Front. Microbiol..

[19] Shi M., Lin X.D., Tian J.H., Chen L.J., Chen X., Li C.X., Qin X.C., Li J., Cao J.P., Eden J.S. (2016). Redefining the invertebrate RNA virosphere. Nature.

[20] Li C.X., Shi M., Tian J.H., Lin X.D., Kang Y.J., Chen L.J., Qin X.C., Xu J., Holmes E.C., Zhang Y.Z. (2015). Unprecedented genomic diversity of RNA viruses in arthropods reveals the ancestry of negative-sense RNA viruses. elife.

[21] Grabherr M.G., Haas B.J., Yassour M., Levin J.Z., Thompson D.A., Amit I., Adiconis X., Fan L., Raychowdhury R., Zeng Q. (2011). Full-length transcriptome assembly from RNA-Seq data without a reference genome. Nat. Biotechnol..

[22] Li J., Andika I.B., Shen J., Lv Y., Ji Y., Sun L., Chen J. (2013). Characterization of rice black-streaked dwarf virus- and rice stripe virus-derived siRNAs in singly and doubly infected insect vector *Laodelphax Striatellus*. PLoS ONE.

[23] Langmead B., Salzberg S.L. (2012). Fast gapped-read alignment with Bowtie 2. Nat. Methods.

[24] Li H., Handsaker B., Wysoker A., Fennell T., Ruan J., Homer N., Marth G., Abecasis G., Durbin R. (2009). The Sequence Alignment/Map format and SAMtools. Bioinformatics.

[25] Thorvaldsdottir H., Robinson J.T., Mesirov J.P. (2013). Integrative Genomics Viewer (IGV): High-performance genomics data visualization and exploration. Brief. Bioinform..

[26] Marchler-Bauer A., Bo Y., Han L., He J., Lanczycki C.J., Lu S., Chitsaz F., Derbyshire M.K., Geer R.C., Gonzales N.R. (2017). CDD/SPARCLE: Functional classification of proteins via subfamily domain architectures. Nucleic Acids Res..

[27] Langmead B., Trapnell C., Pop M., Salzberg S.L. (2009). Ultrafast and memory-efficient alignment of short DNA sequences to the human genome. Genome Biol..

[28] Edgar R.C. (2004). MUSCLE: Multiple sequence alignment with high accuracy and high throughput. Nucleic Acids Res..

[29] Kumar S., Stecher G., Li M., Knyaz C., Tamura K. (2018). MEGA X: Molecular Evolutionary Genetics Analysis across Computing Platforms. Mol. Biol. Evol..

[30] Ding S.W., Voinnet O. (2007). Antiviral immunity directed by small RNAs. Cell.

[31] Mi S., Cai T., Hu Y., Chen Y., Hodges E., Ni F., Wu L., Li S., Zhou H., Long C. (2008). Sorting of Small RNAs into *Arabidopsis* Argonaute Complexes Is Directed by the 5′ Terminal Nucleotide. Cell.

[32] Debat H.J. (2017). An RNA Virome Associated to the Golden Orb-Weaver Spider *Nephila clavipes*. Front. Microbiol..

[33] Ye Z.X., Huang H.J., Lu G., Zhuo J.C., Chen J.P., Zhang C.X., Li J.M. Virome analysis of whitefly collected from Anhui, China.

[34] Ohlund P., Hayer J., Lunden H., Hesson J.C., Blomstrom A.L. (2019). Viromics Reveal a Number of Novel RNA Viruses in Swedish Mosquitoes. Viruses.

[35] Webster C.L., Longdon B., Lewis S.H., Obbard D.J. (2016). Twenty-Five New Viruses Associated with the *Drosophilidae* (Diptera). Evol. Bioinform..

[36] Shi M., Neville P., Nicholson J., Eden J.S., Imrie A., Holmes E.C. (2017). High-Resolution Metatranscriptomics Reveals the Ecological Dynamics of Mosquito-Associated RNA Viruses in Western Australia. J. Virol..

[37] Geib S.M., Calla B., Hall B., Hou S., Manoukis N.C. (2014). Characterizing the developmental transcriptome of the oriental fruit fly, *Bactrocera dorsalis* (Diptera: Tephritidae) through comparative genomic analysis with *Drosophila melanogaster* utilizing modENCODE datasets. BMC Genom..

[38] Calla B., Hall B., Hou S., Geib S.M. (2014). A genomic perspective to assessing quality of mass-reared SIT flies used in Mediterranean fruit fly (*Ceratitis capitata*) eradication in California. BMC Genom..

[39] Medd N.C., Fellous S., Waldron F.M., Xuereb A., Nakai M., Cross J.V., Obbard D.J. (2018). The virome of *Drosophila suzukii*, an invasive pest of soft fruit. Virus Evol..

[40] Schwarz A., Medrano-Mercado N., Schaub G.A., Struchiner C.J., Bargues M.D., Levy M.Z., Ribeiro J.M. (2014). An updated insight into the Sialotranscriptome of *Triatoma infestans*: Developmental stage and geographic variations. PLoS Negl. Trop. Dis..

[41] Feng Y., Krueger E.N., Liu S., Dorman K., Bonning B.C., Miller W.A. (2017). Discovery of known and novel viral genomes in soybean aphid by deep sequencing. Phytobiomes.

[42] Li X., Xu P., Yang X., Yuan H., Chen L., Lu Y. (2017). The genome sequence of a novel RNA virus in *Adelphocoris suturalis*. Arch. Virol..

[43] Al-Wathiqui N., Fallon T.R., South A., Weng J.K., Lewis S.M. (2016). Molecular characterization of firefly nuptial gifts: A multi-omics approach sheds light on postcopulatory sexual selection. Sci. Rep..

[44] De Miranda J.R., Hedman H., Onorati P., Stephan J., Karlberg O., Bylund H., Terenius O. (2017). Characterization of a novel RNA virus discovered in the autumnal moth *Epirrita autumnata* in Sweden. Viruses.

[45] Roundy C.M., Azar S.R., Rossi S.L., Weaver S.C., Vasilakis N. (2017). Insect-Specific Viruses: A Historical Overview and Recent Developments. Adv. Virus Res..

[46] Kondo H., Chiba S., Maruyama K., Andika I.B., Suzuki N. (2019). A novel insect-infecting virga/nege-like virus group and its pervasive endogenization into insect genomes. Virus Res..

[47] Lenz O., Pribylova J., Franova J., Koloniuk I. (2020). Fragaria vesca-associated virus 1, a new virus related to negeviruses. Arch. Virol..

[48] Moreira L.A., Iturbe-Ormaetxe I., Jeffery J.A., Lu G., Pyke A.T., Hedges L.M., Rocha B.C., Hall-Mendelin S., Day A., Riegler M. (2009). A Wolbachia symbiont in *Aedes aegypti* limits infection with dengue, Chikungunya, and Plasmodium. Cell.

[49] Rances E., Ye Y.H., Woolfit M., McGraw E.A., O’Neill S.L. (2012). The relative importance of innate immune priming in Wolbachia-mediated dengue interference. PLoS Pathog.

[50] Hobson-Peters J., Yam A.W.Y., Lu J.W.F., Setoh Y.X., May F.J., Kurucz N., Walsh S., Prow N.A., Davis S.S., Weir R. (2013). A new insect-specific flavivirus from northern Australia suppresses replication of West Nile virus and Murray Valley encephalitis virus in co-infected mosquito cells. PLoS ONE.

[51] Kenney J.L., Solberg O.D., Langevin S.A., Brault A.C. (2014). Characterization of a novel insect-specific flavivirus from Brazil: Potential for inhibition of infection of arthropod cells with medically important flaviviruses. J. Gen. Virol..

