# Supplementary material for: Discovery of Two Novel Negeviruses in a Dungfly Collected from the Arctic
Source: Viruses. 2020 Jun 27;12(7):692. doi: 10.3390/v12070692 (PMC7412485; doi:10.3390/v12070692)
Supplement: Supplementary file 1 [file viruses-12-00692-s001.zip › suppl/Supplementary Table 1.docx]

**Supplementary Table 1. Representative negeviruses used for the identification of nege-like viral contigs**

| **Virus** | **NCBI Accession No.** | **Host** | **Country** |
| --- | --- | --- | --- |
| Loreto virus | NC_034158.1 | *Anopheles albimanus* | Peru |
| Big Cypress virus | NC_034152.1 | *Anopheles atropos* | USA |
| Culex negev-like virus 2 | NC_035123.1 | *Culex australicus* | Australia |
| Piura virus | NC_034155.1 | *Culex* sp. | Colombia |
| Castlerea virus | NC_034569.1 | *Anopheles*, *Culex*, and *Aedes* mosquitoes | Australia |
| Negev virus | NC_030294.1 | *Culex vishunui* | Philippines |
| Ying Kou virus | NC_040636.1 | *Culex pipiens* | China |
| Brejeira virus | NC_030743.1 | *Culex* sp. | Brazil |
| San Bernardo virus | NC_034154.1 | Mosquito | Colombia |
| Daeseongdong virus 1 | NC_028487.1 | *Culex pipiens* | South Korea |
| Tanay virus | NC_024071.1 | *Culex quinquefasciatus* | Philippines |
| Biratnagar virus | NC_034153.1 | Mosquito | Nepal |
| Wallerfield virus | NC_023440.1 | *Culex portesi* | Trinidad and Tobago |
| Goutanap virus | NC_025357.1 | *Culex nebulosus* | Cote d'Ivoire |
| Biggievirus Mos11 | MH603566.1 | *Culex* sp. | India |
| Uxmal virus | MH719095.1 | *Ochlerotatus taeniorhynchus* | Mexico |
| Santana virus | JQ675606.1 | *Culex* sp. | Brazil |
| Bustos virus | LC103139.1 | *Mansonia* sp. | Philippines |
