# Supplementary material for: Discovery of Two Novel Negeviruses in a Dungfly Collected from the Arctic
Source: Viruses. 2020 Jun 27;12(7):692. doi: 10.3390/v12070692 (PMC7412485; doi:10.3390/v12070692)
Supplement: Supplementary file 1 [file viruses-12-00692-s001.zip › suppl/Supplementary Table 2.docx]

**Supplementary Table 2. Primers used in this study^#^**

| **Primer** | **Sequence (5' to 3')** | **Purpose** |
| --- | --- | --- |
| Long primer | CTAATACGACTCACTATAGGGCAAGCAGTGGTATCAACGCAGAGT | Amplification of 5′/3′ RACE fragment |
| Short primer | CTAATACGACTCACTATAGGGC |  |
| 5′-RACE GSP1 | CAGCGTCATAGCCTGACGTTGG | Amplification of 5′ RACE fragment of NVD1 |
| 3′-RACE GSP1 | GCTTCTCAATCTGATAATACCG | Amplification of 3′ RACE fragment of NVD1 |
| NVD1-F | GATATATCAAACAAACTAATGTC | Amplification of NVD1 viral genome |
| NVD1-R | GTCTATAAGATAAAGAAAAT |  |
| 5′-RACE GSP2 | GCGTCTAGAAGATCATAGCATCTG | Amplification of 5′ RACE fragment of SVD1 |
| 3′-RACE GSP2 | TCCGTATAAGCGATTCACTTCTG | Amplification of 3′ RACE fragment of SVD1 |
| SVD1-F | GAATTTAATTACAACAACCTTTC | Amplification of NVD1 viral genome |
| SVD1-R | GTCGATTAATTAAAATAAAAG |  |

^#^ NVD1: Nelorpivirus dungfly1; SVD1: Sandewavirus dungfly1;
